# Foley Catheter versus Vaginal Misoprostol for Labour Induction

**DOI:** 10.1155/2015/845735

**Published:** 2015-10-18

**Authors:** Nasreen Noor, Mehkat Ansari, S. Manazir Ali, Shazia Parveen

**Affiliations:** Department of Obstetrics and Gynaecology and Department of Pediatrics, JNMCH, AMU, L-Block 107 Safina Apartment, Medical Road, Aligarh 202002, India

## Abstract

*Objectives*. To compare the efficacy and safety of intravaginal misoprostol with transcervical Foley catheter for labour induction. *Material and Methods*. One hundred and four women with term gestation, with Bishop score < 4, and with various indications for labour induction were randomly divided into two groups. In Group I, 25 *μ*g of misoprostol tablet was placed intravaginally, 4 hourly up to maximum 6 doses. In Group II, Foley catheter 16F was placed through the internal os of the cervix under aseptic condition and then inflated with 50 cc of sterile saline. Statistical analysis was done using SPSS software. * Results*. The induction to delivery interval was 14.03 ± 7.61 hours versus 18.40 ± 8.02 hours (*p* < 0.01). The rate of vaginal delivery was 76.7% versus 56.8% in misoprostol and transcervical Foley catheter group, respectively. Uterine hyperstimulation was more common with misoprostol. Neonatal outcome was similar in both the groups. *Conclusion*. Intravaginal misoprostol is associated with a shorter induction to delivery interval as compared to Foley's catheter and it increases the rate of vaginal delivery in cases of unripe cervix at term. Transcervical Foley catheter is associated with a lower incidence of uterine hyperstimulation during labour.

## 1. Introduction

In the recent decade, there has been a considerable increase in the rate of labour induction. Achievement of a vaginal delivery for a woman who requires induction of labour may be among the greatest challenges facing obstetricians today. Labour induction is usually performed when the risks of continuing a pregnancy are more than the benefits of delivery. Indications for induction of labour include immediate conditions such as severe preeclampsia or ruptured membranes with chorioamnionitis. The other common medical and obstetric indications include membrane rupture without labour, gestational hypertension, postdated pregnancy, oligohydramnios, nonreassuring fetal status, intrauterine growth restriction, chronic hypertension, and diabetes [1]. Undoubtedly, cervical ripening has a close relationship with the success rate of vaginal delivery. Different methods are used for labour induction but none of the available methods of induction of labour is free of associated medical risks; therefore, labour should only be induced when the risk of allowing the continuation of pregnancy outweighs the risk of induction. Ideally, agents used for induction should mimic spontaneous labour without causing excessive uterine activity. The most common methods of labour induction when the status of cervix is unfavourable involve intravaginal use of misoprostol, transcervical insertion of Foley's catheter, and insertion of prostaglandin gel whereas with a ripe cervix oxytocin may be administered intravenously. Serum levels after vaginal absorption are more prolonged; irrespective of serum levels, vaginally absorbed misoprostol has locally mediated effects; thus there has been increasing interest in misoprostol for use as a pharmacological agent for labour induction. However, there remains some controversy concerning the dosage, the mode, and interval of administration of misoprostol. Although perhaps more effective, use of a high dose could be associated with an increased risk for hyperstimulation of the uterus; however there are ongoing trials regarding optimal dose, dosing regimen, and route of administration. In the case of women who have previously undergone a caesarean section and thereby run an increased risk for uterine rupture in connection with vaginal delivery, induction of labour with misoprostol may further enhance this risk and is not recommended. Another procedure adopted for routine induction of labour involves transcervical application of Foley's catheter. Such a catheter appears to induce labour not only through direct mechanical dilation of the cervix but also by stimulating endogenous release of prostaglandins. The aim of this study is the comparison of vaginal misoprostol and transcervical Foley's catheter for induction of labour.

## 2. Material and Methods

This randomized clinical study was conducted in the Department of Obstetrics and Gynaecology in collaboration with the Department of Paediatrics, JNMCH, AMU, Aligarh (UP), India, during May 2013–August 2014. The included criteria were singleton pregnancy cephalic presentation, gestation age >37 weeks on the basis of LMP or first trimester ultrasonography, intact membranes, unfavourable cervix (Bishop score ≤ 4), and imminent delivery for fetal or maternal indication. Women were excluded from the study if any of the following criteria were encountered: rupture of membranes, chorioamnionitis, antepartum haemorrhage, cervical dilation >2.5 cm, temperature >38°C, contracted pelvis, fetal distress, polyhydramnios, indication for immediate delivery, and previous caesarean section or other uterine surgeries (for Group I).

A total of one hundred and four (104) women requiring indicated induction of labour with an unfavourable cervix (Bishop score ≤ 4) were included in the study. They were randomly divided into two groups: 60 women induced with intravaginal misoprostol (Group I) and 44 women induced with transcervical Foley catheter (Group II). At first, the method of the study was completely explained to them; if the written consent was obtained, they were enrolled in the study. This study was approved by the Ethics Committee of Faculty of Medicine, Aligarh Muslim University. Cases were selected from antenatal clinic (ANC), outpatient department (OPD), and patients admitted in the hospital. The two groups were comparable with respect to maternal age, parity, and gestational and preinduction Bishop score. Demographic and clinical data were collected at routine antenatal visits. In Group I, 25 mcg of misoprostol tablet was placed intravaginally, 4 hourly for maximum 6 doses. In the presence of spontaneous and frequent contractions (>40–45 seconds every 3 minutes), the next dose was not administered. If there was no effective uterine contractions after the sixth dose, then it was considered as failure of induction by the concerned method. In Group II, 18 F Foley catheter was inserted into the endocervical canal under direct vision by doing a per-speculum examination. The catheter was advanced into the endocervical canal. Once past the internal os, the balloon was filled with 50 mL of sterile saline solution and the catheter was taped to the inner thigh to maintain traction. The catheter was checked for extrusion of the balloon from the cervix every 6 hours by cervical examination and the catheter remained in place until the balloon was expelled spontaneously and labour augmentation was done by artificial membrane rupture or oxytocin drip (2.5 or 5 IU in 500 mL of Ringer's lactate solution was started then and it was titrated according to frequency and intensity of uterine contractions) whichever is indicated. The primary outcome measures were induction to delivery interval and secondary outcome measures include uterine contractile abnormalities like uterine tachysystole (6 contractions in a 10-minute period), uterine hypertonus (a single contraction lasting longer than 2 minutes) and uterine hyperstimulation is when either condition leads to a nonreassuring fetal heart rate pattern, meconium stained liquor, mode of delivery, maternal and neonatal outcome, neonatal birth weight, and Apgar score. Any maternal or fetal complications were also recorded.

## 3. Results

A total of one hundred and four (104) women were included in the study. They were randomly divided into two groups: Group I: women induced with intravaginal misoprostol (*n* = 60) and Group II: women induced with transcervical Foley catheter (*n* = 44). Maternal baseline characteristics were similar between the two groups in terms of age, parity, gestational age, preinduction Bishop score, and indications for induction (Table 1).

As shown in Table 2 the induction to delivery interval (mean ± SD) in women induced with intravaginal misoprostol was 14.03 ± 7.61 hours while that of women induced with transcervical Foley catheter was 18.40 ± 8.02 hours. The induction to delivery interval in misoprostol group was significantly shorter than that in Foley catheter group (*p* < 0.01).

The use of oxytocin and ARM for labour augmentation was significantly higher in women induced with Foley catheter as compared to women induced with intravaginal misoprostol 77.2% versus 48.3% and 95.5% versus 66.7%, respectively. Combined use of oxytocin and ARM was 41.7% and 77.2% in misoprostol and Foley catheter group, respectively, and statistically it was very highly significant (*p* < 0.001). Uterine contractile abnormalities like hyperstimulation were reported in 11.7% of women while there was no case of hyperstimulation noted in Foley catheter group (Table 3).

As depicted in Table 4, the rate of vaginal delivery and caesarean section was 76.7% versus 56.8% and 23.3% versus 43.2% in misoprostol and Foley catheter group, respectively. The rate of vaginal delivery was significantly more in misoprostol group as compared to Foley catheter group (*p* < 0.05). In this study, there was a tendency towards more frequent caesarean section in response to fetal distress among women who were given misoprostol. This finding is in agreement with most of the studies that have demonstrated a higher incidence of hyperstimulation associated with fetal distress in women induced with misoprostol. In women induced with Foley catheter, nonprogression of labour and scar tenderness were seen in 20.5% and 9.1% women, respectively. Meconium amniotic fluid was seen in 5 women (8.3%) induced with misoprostol and 4 women (9.1%) induced with Foley catheter. Both the groups were comparable in terms of meconium amniotic fluid as an indication of caesarean section. The caesarean section rate was more in Foley catheter group as compared to misoprostol group and the results were statistically significant (*p* < 0.05).

The birth weight (mean ± SD) was 2.79 ± 0.43 kg and 2.91 ± 0.53 kg in misoprostol and Foley catheter group. The difference in the birth weight between the two study groups was statistically not significant (*p* > 0.05). The Apgar score at 1 minute and 5 minutes (mean ± SD) was 7.80 ± 0.77 versus 7.91 ± 0.33 and 8.92 ± 0.38 versus 8.98 ± 0.15 in misoprostol and Foley catheter group, respectively (Table 5). Statistically there was no significant difference in the Apgar score between the two groups at 1 minute and 5 minutes (*p* > 0.05).

## 4. Discussion

Induction of labour is an integral component of all maternity practice and is often taken up in the interest of the mother and the fetus. Labour induction in the presence of an unfavorable cervix is associated with an increased likelihood of prolonged labour and increased incidence of caesarean section. Hence, the use of cervical ripening agents prior to conventional methods of induction is now a standard practice. Until now different methods for labour induction are used. In literature, contradictory results are reported regarding efficacy and safety of the induction methods. Therefore in this study, we compared the efficacy and safety of 25 *μ*g vaginal misoprostol with transcervical Foley catheter for induction of labour.

Our results on induction to delivery interval show that the interval was significantly shorter in misoprostol group as compared to Foley catheter group. Our findings were similar to Promila et al. [2], Sheikher et al. [3], Filho et al. [4], and Roudsari et al. [5], who also found significantly shorter induction to delivery interval in misoprostol group. Tuuli et al. [6] reported that the total duration of labour was not significantly different in women induced with misoprostol compared with the Foley catheter (median duration from 1 to 10 cm: 12 versus 14.2 hours, *p* = 0.19). Jindal et al. [7] also reported shorter interval for misoprostol compared to Foley's catheter (11.58 hours versus 19.45 hours). The shorter induction delivery interval in misoprostol group could be explained on the basis of greater oxytocic effect on uterus via vaginal route due to direct access to myometrium by cervical canal. In the study performed by Chung et al. [8] and Adeniji et al. [9], the induction to delivery interval did not differ significantly between the two groups. Our study is not in accordance with Prager et al. [10], who found that induction to delivery interval was significantly shorter in Foley catheter group as compared to misoprostol and PGE2; the most important cause for this may be lower dose of misoprostol (25 *μ*g) used in our study compared with their studies [10]. Use of oxytocin for labour augmentation was significantly higher in women induced with Foley catheter as compared to women induced with intravaginal misoprostol. Uterine contractile abnormalities were more common in women using misoprostol as compared to Foley's catheter. The finding that transcervical Foley catheter is associated with no risk of hyperstimulation may be particularly useful when inducing labour in woman with previous caesarean section who are at increased risk of uterine rupture. No case of tachysystole or uterine rupture was found in both the groups. Roudsari et al. [5] found hyperstimulation occurring more frequently in the misoprostol group. Chung et al. [8] in their study found that hyperstimulation occurred in 33.3% women in misoprostol group and 11.1% women in Foley catheter group. Mozurkewich et al. [1] found that contractile abnormalities were more frequent in the misoprostol group than the Foley catheter group and thus this finding is in agreement with the findings that have demonstrated a higher incidence of hyperstimulation associated with fetal distress in women induced with misoprostol. Both the groups were comparable in terms of meconium amniotic fluid as an indication of caesarean section. Statistically there was no significant difference in the Apgar score between the two groups at 1 minute and 5 minutes. Similar results were obtained by Filho et al. [4] and Roudsari et al. [5] and our present study supports these results.

## 5. Conclusion

The present study suggests intravaginal misoprostol is associated with a shorter induction to delivery interval as compared to Foley's catheter and it increases the rate of vaginal delivery in cases of unripe cervix at term. Transcervical Foley catheter is associated with a lower incidence of uterine hyperstimulation; thus Foley catheter may be a reasonable alternative for patients who are at risk of uterine rupture during labour.

## Figures and Tables

**Table 1:** Demographic profile and indication for induction.

Parameters	Group I (*n* = 60) (misoprostol)	Group II (*n* = 44) (Foley catheter)	“*p*” value
Age (years) (mean ± SD)	25.1 ± 2.8	25.6 ± 4.1	>0.05
Gravidity			
Primigravida	41.7%	31.8%	>0.05
Multigravida	58.3%	68.2%	>0.05
Gestational age (weeks) (mean ± SD)	39.1 ± 1.4	39.4 ± 1.2	>0.05
Indication for induction			
Oligohydramnios	11 (18.3)	08 (18.2)	>0.05
Preeclampsia	11 (18.3)	04 (09.1)	>0.05
Intrauterine growth rsestriction	07 (11.7)	04 (09.1)	>0.05
Gestational diabetes mellitus	02 (03.4)	01 (02.3)	>0.05

**Table 2:** Induction to delivery interval (mean ± SD).

Parameters	Group I (*n* = 60) (misoprostol)	Group II (*n* = 44) (Foley catheter)	“*p*” value
Induction to active phase interval (hrs) (mean ± SD)	11.6 ± 5.21	11.8 ± 5.82	>0.05
Induction to delivery interval (hrs) (mean ± SD)	14.03 ± 7.61	18.40 ± 8.02	<0.01

**Table 3:** Outcome in labour.

	Group I		Group II			
Augmentation required	(Misoprostol)		(Foley catheter)		*X* ^2^	*p* value
	*n*	%	*n*	%		
Oxytocin drip	29	48.3	34	77.2	8.9	<0.01
Artificial rupture of membrane	40	66.7	42	95.5	12.6	<0.001
Oxytocin + ARM	25	41.7	34	77.2	13.1	<0.001
*Complications*						
Hyperstimulation	07	11.7	00	00.0	—	—
Tachysystole	00	00.0	00	00.0	—	—
Uterine rupture	00	00.0	00	00.0	—	—

**Table 4:** Comparison of mode of delivery.

	Group I		Group II				
Mode of delivery	(Misoprostol)		(Foley catheter)		Total		“*p*” value
	*n*	%	*n*	%	*n*	%	
Vaginal delivery	46	76.7	25	56.8	71	68.3	<0.05
Caesarean delivery	14	23.3	19	43.2	33	31.7	<0.05
Total	60	100.0	44	100.0	104	100.0	

**Table 5:** Neonatal outcome in Group I and Group II.

Parameters	Group I (*n* = 60) (misoprostol)	Group II (*n* = 44) (Foley catheter)	“*p*” value
Birth weight (kg) (mean ± SD)	2.79 ± 0.43	2.91 ± 0.53	>0.05

Apgar score (at 1 min) Mean ± SD	7.80 ± 0.77	7.91 ± 0.33	>0.05

Apgar score (at 5 min) Mean ± SD	8.92 ± 0.38	8.98 ± 0.15	>0.05

Admission in neonatal intensive care unit	13.3%	13.6%	>0.05

Meconium aspiration syndrome	8.3%	9.1%	>0.05

## References

[1] Mozurkewich E., Chilimigras J., Koepke E., Keeton K., King V. J. (2009). Indications for induction of labour: a best-evidence review. *BJOG: An International Journal of Obstetrics and Gynaecology*.

[2] Promila J., Kaur G. B., Bala T. (2007). A comparison of vaginal misoprostol versus Foley's catheter with oxytocin for induction of labor. *The Journal of Obstetrics and Gynecology of India*.

[3] Sheikher C., Suri N., Kholi U. (2009). Comparative evaluation of oral misoprostol, vaginal misoprostol and intracervical Folley's catheter for induction of labour at term. *JK Science*.

[4] Filho O. B. M., Albuquerque R. M., Cecatti J. G. (2010). A randomized controlled trial comparing vaginal misoprostol versus foley catheter plus oxytocin for labor induction. *Acta Obstetricia et Gynecologica Scandinavica*.

[5] Roudsari F. V., Ayati S., Ghasemi M. (2011). Comparison of vaginal misoprostol with Foley catheter for cervical ripening and induction of labor. *Iranian Journal of Pharmaceutical Research*.

[6] Tuuli M. G., Keegan M. B., Odibo A. O., Roehl K., Macones G. A., Cahill A. G. (2013). Progress of labor in women induced with misoprostol versus the Foley catheter. *American Journal of Obstetrics and Gynecology*.

[7] Jindal P., Gill B. K., Tirath B. (2007). A comparison of vaginal misoprostol versus Foley's catheter with oxytocin for induction of labour. *The Journal of Obstetrics and Gynecology of India*.

[8] Chung J. H., Huang W. H., Rumney P. J., Garite T. J., Nageotte M. P. (2003). A prospective randomized controlled trial that compared misoprostol, foley catheter, and combination misoprostol-foley catheter for labor induction. *American Journal of Obstetrics and Gynecology*.

[9] Adeniji A. O., Olayemi O., Odukogbe A. A. (2006). Intravaginal misoprostol versus transcervical Foley catheter in pre-induction cervical ripening. *International Journal of Gynecology and Obstetrics*.

[10] Prager M., Eneroth-Grimfors E., Edlund M., Marions L. (2008). A randomised controlled trial of intravaginal dinoprostone, intravaginal misoprostol and transcervical balloon catheter for labour induction. *BJOG: An International Journal of Obstetrics & Gynaecology*.

